# The Role of the Medial Habenula Cholinergic System in Addiction and Emotion-Associated Behaviors

**DOI:** 10.3389/fpsyt.2019.00100

**Published:** 2019-02-28

**Authors:** Hyun Woo Lee, Soo Hyun Yang, Jin Yong Kim, Hyun Kim

**Affiliations:** Department of Anatomy, College of Medicine, Korea University, Seoul, South Korea

**Keywords:** habenula, cholinergic system, nicotine addiction and withdrawal, drug addiction, fear, depression

## Abstract

The habenula is a complex nucleus composed of lateral and medial subnuclei, which connect between the limbic forebrain and midbrain. Over the past few years, the lateral habenula has received considerable attention because of its potential roles in cognition and in the pathogenesis of various psychiatric disorders. Unlike extensively studied lateral habenula, anatomically and histologically distinct medial habenula remains largely understudied. The medial habenula can be further subdivided into a dorsal region containing excitatory neurons that express the tachykinin neuropeptide substance P and a ventral region containing dense cholinergic neurons. Although the medial habenula is the source of one of the major cholinergic pathways in the brain, relatively few studies have been conducted to understand its roles. Recently, however, the medial habenula cholinergic system has attracted more attention because of its potential to provide therapeutic targets for the treatment of nicotine withdrawal symptoms, drug addiction, and various mood disorders. Here, we discuss the role of the medial habenula cholinergic system in brain function.

## Introduction

The habenula is part of the epithalamus and is divided into medial and lateral subregions, each with a unique gene-expression profile (1). Over the last few years, studies have shown that neuronal activity in the lateral habenula (LHb) is regulated by factors in the external environment, such as rewards or aversive stimuli, and this altered LHb neuronal activity is associated with the onset of depression (2–4). The medial habenula (MHb) is divided into two subnuclei on the basis of cell type. Cholinergic neurons are located in the ventral two-thirds of the MHb (MHbV), and substance P-ergic neurons are located exclusively in the dorsal part of the MHb (MHbD) (5). Structurally, the MHbD receives input from the bed nucleus of the anterior commissure (BAC) and innervates the outermost portion of the interpeduncular nucleus (IPN). By contrast, the MHbV receives neural input from the triangular septum (TS) and projects to the central part of the IPN (6, 7). The function of substance P-ergic signaling in MHbD neurons is not well known, but MHbD-specific lesion and optogenetic studies have reported that MHbD mediates exercise motivation, regulates the hedonic state, and supports primary reinforcement (8, 9). Signaling from the MHbV cholinergic neurons is involved in drug addiction, including nicotine addiction, nicotine withdrawal symptoms, anxiety, and depression. In this review, we will discuss how cholinergic signaling in the MHbV–IPN pathway affects various brain functions. Table 1 briefly summarizes the results of studies investigating the function of medial habenula cholinergic signaling in addiction and mood-related behaviors.

**Table 1:** Psychological symptoms related to habenular cholinergic signaling.

**Psychological symptoms**	**Experimental methods**	**Results**	**References**
Nicotine withdrawal symptoms	•Nicotine cessation after chronic exposure	•Activation of IPN GABAergic neurons	(10)
	•Optogenetic stimulation of IPN GABAergic neurons	•Physical withdrawal symptoms	(10)
	•Re-exposure to nicotine after chronic administration •Administration of mecamylamine or corticotropin releasing factor into IPN during nicotine withdrawal	•Increased spontaneous firing of habenula cholinergic neurons •Increased nicotine withdrawal-induced anxiety	(11) (12)
Drug addiction	•Chronic morphine administration	•Reduced c-fos immunoreactivity	(13)
	•Chronic exposure to D-amphetamine, methamphetamine, MDMA, and cocaine	•Axonal neurodegeneration of MHb neurons	(14, 15)
	•Ibogaine treatment in rodents (therapeutic effect on drug addiction)	•Reduced self-administration of cocaine, alcohol, and morphine	(16–18)
Fear	•Ablation of bed nucleus of the anterior commissure (BAC)	•Increased fear response	(6)
	•Ablation of habenula cholinergic neurons and genetic inactivation of GABA_B_ receptors	•Enhanced expression of fear	(19)
	•Conditional deletion of cannabinoid receptor 1 from MHb neurons	•Reduced fear-conditioned freezing	(20)
Depression	•Knockdown of habenula ChAT gene expression	•Anhedonia-like behavior	(21)
	•Habenula cholinergic gene expression in rat model of depression and humans with major depressive disorder	•Decreased expression of cholinergic signaling genes in the habenula	(21)
	•Electrical lesion of MHb in the CUMS-exposed rats	•The lower hedonic state induced by CUMS was restored	(22)

## Basic Characteristics of Habenular Cholinergic Neurons

Habenula cholinergic neurons are the source of a major cholinergic pathway in the central nervous system, and innervate the IPN neurons via the fasciculus retroflexus (fr). Since choline acetyltransferase (ChAT) is the only enzyme responsible for the biosynthesis of acetylcholine (ACh) (23), it is frequently used as a marker of cholinergic neurons. Immunohistochemistry with an anti-ChAT antibody and *in situ* hybridization against ChAT mRNA both show that habenula cholinergic neurons are restricted to the MHbV (5, 24). Habenula cholinergic neurons release two neurotransmitters, glutamate and ACh, as demonstrated by the colocalization of the vesicular acetylcholine transporter (VAChT) and vesicular glutamate transporter 1 (VGLUT1), visualized with immunogold electron microscopy (25), and by optogenetic stimulation in ChAT-ChR2-EYFP transgenic mice, in which cholinergic neurons express Channelrhodopsoin-2 (ChR2) (26). According to this optogenetic study, the short photostimulation of habenula cholinergic neurons produces ionotropic glutamate receptor-mediated fast excitatory postsynaptic currents, whereas tetanic photostimulation evokes nicotinic acetylcholine receptor (AChR)-mediated slow inward currents in the IPN neurons. Cholinergic habenula neurons exhibit pacemaker activity under the control of circadian rhythms and nicotine withdrawal. Spontaneous firing in the MHb is higher during the day than during the night, probably because of the expression of a circadian gene (27, 28). Although it is not known whether the expression of cholinergic genes is actually involved in generating the circadian rhythm, the MHb is more rhythmic than the LHb (29).

## Nicotine Addiction and Withdrawal

Genome-wide association studies suggest that specific single-nucleotide polymorphisms associated with an increased risk of nicotine dependence and nicotine addiction are located within a specific gene cluster on human chromosome 15 that encodes the α5, α3, and β4 nAChR subunits (30–34). Since α3, α5, and β4 nAChR subunits are enriched in the MHbV–IPN pathway, it is has been suggested that this pathway may play a critical role in nicotine withdrawal behaviors (35, 36). The functional nAChR channel forming α3 and β4 subunits are mainly expressed in the MHbV, and α5 subunit is highly expressed in the IPN (37–39). After chronic administration of nicotine in mice, nicotine cessation results in withdrawal symptoms. This behavioral effect can be reproduced by injecting mecamylamine, a non-selective nAChR antagonist, into the IPN of mice chronically exposed to nicotine. Nicotine cessation and mecamylamine administration activate GABAergic neurons in the IPN, leading to physical nicotine withdrawal symptoms (10). Optogenetic stimulation of GAD2-expressing GABAergic neurons in the IPN induces physical withdrawal symptoms in both nicotine-naïve and chronic nicotine-exposed mice, and the affective symptoms are ameliorated by IPN-selective infusion of a NMDA receptor antagonist (10). Therefore, glutamate release from MHb neurons innervating the IPN is necessary for somatic manifestation of nicotine withdrawal. During chronic nicotine exposure, the expression of nAChRs containing the β4-subunit is upregulated in somatostatin-positive GABAergic neurons in the IPN. Selective blockade of these β4-subunit-containing nAChRs induces more dramatic somatic withdrawal signs in nicotine-exposed mice than nicotine-naïve mice (10). Because somatostatin-positive GABAergic neurons in the IPN project principally to the median raphe/paramedian raphe and dorsal tegmental area, two regions rich in serotonergic neurons (39), the activation of these IPN GABAergic neurons by nicotine withdrawal may modulate downstream serotonin release. Zhao-Shea et al. presented a new more complex mechanism for nicotine withdrawal-induced anxiety-related behavior involving corticotropin releasing factor (CRF) signaling from the VTA to the IPN (12). After chronic nicotine administration, CRF synthesis is upregulated in the VTA dopaminergic neurons and the level of the CRF receptor 1 is also increased in a particular subnucleus of the ventral IPN, which appears to receive efferent axons from the VTA. CRF secreted by the VTA may stimulate the neuronal activity of the IPN by promoting glutamate release through the CRF receptor 1 during chronic nicotine withdrawal. Blockade of the CRF receptor by an antagonist in the IPN or CRF knockdown in the VTA was shown to alleviate the anxiety induced by nicotine withdrawal.

IPN is a complex structure composed of several subnuclei (40, 41) and cholinergic signaling in the MHb-IPN pathway has been reported to be mediated by α5 nAChR subunit-expressing GABAergic neurons in the IPN, which project principally to the median/paramedian raphe and dorsal tegmental area (39). Morton *et al*. demonstrated that acetylcholine and nicotine-evoked responses in the IPN neurons were markedly reduced in α5-null mice (42). However, unlike the result obtained using broad optogenetic stimulation of GAD2-expressing GABAergic neurons in the IPN, selective optogenetic stimulation of only α5-expressing GABAergic neurons did not elicit the somatic signs associated with nicotine withdrawal and had no effect on locomotion or anxiety (42). Rather, after prior stimulation or exposure to nicotine, optogenetic stimulation of α5-expressing IPN neurons produced aversion. The difference in the effect of optogenetic stimulation between the above two mentioned studies may be off-target effect of GAD2-expressing neurons not being limited to the IPN or the function of the neural circuit formed by other GABAergic neurons that do not express α5.

Many studies have shown that nicotine withdrawal reduces levels of dopamine and serotonin (43, 44). In addition, although there is no change in the nicotine-induced firing rate in habenula cholinergic neurons in mice chronically treated with nicotine, re-exposure to nicotine during withdrawal increases the rate of spontaneous firing. This nicotine-sensitive regulation of pacemaker activity in MHb cholinergic neurons may contribute to smoking relapses (11). Recently, Wolfman et al. reported that a high dose of nicotine induces aversive behavior that is mediated by IPN GABAergic inputs to the laterodorsal tegmentum (LDTg) (45). They showed that selective optogenetic stimulation of IPN axon terminals in the LDTg elicits significant behavioral avoidance in a real-time place-preference test. Similarly, optogenetic inhibition of IPN axon terminals in the LDTg blocks conditioned place aversion in mice that receive an aversive nicotine dose (1.5 mg kg^−1^). Understanding how nicotine cessation and blockade of acetylcholine signaling after chronic nicotine exposure induce activation of GABAergic neurons in the MHb–IPN pathway will require a sophisticated examination of the connections between MHb cholinergic neurons and IPN neurons, the neural circuits within the IPN, and the connections between IPN GABAergic neurons and midbrain serotonergic neurons.

## Drug Addiction

The habenula has also been linked to drug addiction more generally through a series of rodent experiments and human genome-wide association studies (31–33, 43, 46). Genetic variation in the nAChR subunit CHRNA5 is associated with addiction to drugs such as alcohol and cocaine (47, 48); therefore, a number of studies have investigated the association between nAChR expression and drug addiction in habenula cholinergic neurons. Chronic morphine administration in mice (20–100 mg/kg three times per day) decreases acetylcholinesterase activity in the MHb, but activity returns to baseline levels during morphine withdrawal. Chronic morphine administration reduces c-fos activity in the MHb (13), whereas cocaine promotes c-fos expression in the LHb (49, 50). Ibogaine, which has a therapeutic effect on drug addiction, reduces the self-administration of cocaine, alcohol, and morphine in rodents (16–18). Similarly, 18-methoxycoronaridine (18-MC), a derivative of ibogaine, inhibits abuse of morphine, cocaine, and nicotine but without the severe side effects seen with ibogaine treatment (51, 52). Because 18-MC has antagonistic activity against β4-containing nAChRs in the MHb and IPN (53), MHb cholinergic signaling is thought to play a major role in drug addiction. Habenula cholinergic neurons regulate the self-administration of drugs such as cocaine and methamphetamine, as well as the reinstatement of drug-seeking behaviors (2, 44, 53). Chemogenetic activation of habenula cholinergic neurons by a cre-dependent DREADD (Designer Receptors Exclusively Activated by Designer Drugs) system in ChAT-cre mice induces behaviors that mimic drug-seeking behavior, such as the reinstatement of a cocaine-induced conditioned place preference (54).

Nicotine abuse prompts axonal neurodegeneration in the central region of the fr, and this axonal neurodegeneration is also observed following chronic exposure to various other drugs such as D-amphetamine, methamphetamine, MDMA, and cocaine. Nicotine and other drugs induce neurodegeneration of the central core region and external sheath region of the fr (14, 15). A recent study showed that the sheath region surrounding the fr connects the LHb and the ventral tegmental area (VTA) reciprocally, and that the core region inside the fr is an efferent projection from the MHb to the IPN (55). Thus, if different drugs cause different patterns of fr neurodegeneration, the relative modulation of the LHb–VTA and the MHb–IPN pathways may vary.

## Fear

The bed nucleus of the anterior commissure (BAC) and the triangular septum (TS) mainly project to the MHbD and MHbV, respectively (6, 7). Using the immunotoxin-mediated cell targeting method, Yamaguchi et al. selectively ablated BAC and TS neurons (6). BAC-ablated mice showed an increased fear response when freezing was measured after presentation of electric shocks, whereas TS-ablated mice did not. These effects of ablation of projection neurons to the MHb suggest that MHbD is associated with the fear response, but not the MHbV. However, direct ablation of MHbD neurons did not change in the fear response (9).

Notably, direct ablation of MHbV cholinergic neurons and genetic inactivation of GABA_B_ receptors in cholinergic neurons increased the expression of conditioned fear (19). GABA_B_ receptors are abundant in habenula cholinergic neurons, which, as noted above, project to the IPN. Notably, MHbV-derived presynaptic GABA_B_ receptors mediate the release of glutamate in the IPN upon activation by GABA released from IPN GABAergic neurons. This retrograde activation of GABA_B_ receptors promotes prolonged enhancement of glutamate release (56). To fully understand the function of the MHbV cholinergic–IPN GABAergic neuronal pathway, it will be necessary to identify the physiological conditions that stimulate MHb cholinergic neurons and develop a systematic understanding of the mechanisms underlying the interactions between acetylcholine and glutamate release from MHb neurons and GABA release from IPN neurons.

Conditional deletion of cannabinoid type 1 (CB_1_) from MHb neurons by injecting Cre recombinase expressing AAV into the MHb of CB_1_-floxed mice reduced strongly the freezing response in cued and contextual fear conditioning (20). Pharmacological blockade of the CB_1_ receptor in the IPN also reduced fear-conditioned freezing in mice. Consistent with the interpretation that downregulation of cholinergic signaling enhances the fear response (19), genetic deletion of MHb-CB_1_ and pharmacological inhibition of CB_1_R activity enhances cholinergic neurotransmission. Therefore, synaptic modulation of cholinergic neurotransmission in the MHb-to-IPN synapses by presynaptic CB_1_ receptor and GABA_B_ receptor activities derived from the MHb cholinergic neurons plays a regulating role in the expression of fear memory.

## Depression

Cholinergic hyperactivity in the brain has long been associated with depressive phenotypes (57–59). Increased extracellular levels of ACh after administration of acetylcholinesterase inhibitor can lead to depressed mood states in both normal humans and rodents (60, 61). The depression-like behaviors or symptoms are reversed by broad administration of nAChRs or mAChRs antagonists in an established animal model or human patients (62–65). Recent shRNA-mediated knockdown of ChAT in the rat habenula induces anhedonia but not despair-like behavior (21). Downregulation of cholinergic signaling in the habenula, including decreased expression of the genes related to cholinergic signaling, such as ChAT, CHT, VAChT, CHRNA3, and CHRNB4, has been demonstrated in an animal model of depression and in suicide victims diagnosed with major depressive disorder (21). Furthermore, selective pharmacogenetic activation of habenula cholinergic neurons via DREADDs in ChAT-cre mice leads to the excitation of dopamine neurons in the VTA and reduces serotonin immunoreactivity in the DRN (21), suggesting that habenular cholinergic neurons directly or indirectly regulate monoaminergic neurons in the midbrain.

However, Xu et al. reported that electrically-induced lesions of the MHb in rats attenuated the lower sucrose consumption caused by chronic unpredictable mild stress (CUMS), but did not attenuate the increased immobility time in the FST, in accord with the cholinergic hyperactivity theory of depression (22). The release of substance P derived from MHbD was increased in the IPN of CUMS-exposed rats and the resulting higher SP levels increased the neuronal activity of the IPN. This study suggests that hyperactivity of the MHb-IPN pathway triggers the anhedonia-like behavior and higher SP signaling mediates a lower hedonic state. The increased hedonic state caused by the MHb-lesion in the CUMS-exposed rats is contradictory in that both the reduced cholinergic signaling following ChAT knockdown in the MHbV (21) and genetic ablation of MHbD (9) resulted in anhedonia-like behavior. Considering that SP-expressing neurons are present in the MHbD and that the SP receptor Tacr1 is mainly expressed in the MHbV (5), the close correlation between neuronal signaling in the MHbD and MHbV in the MHb-IPN pathway will require further study.

## Topographical Anatomy of the MHbV-IPN Pathway and Further Directions

**Figure 1:**
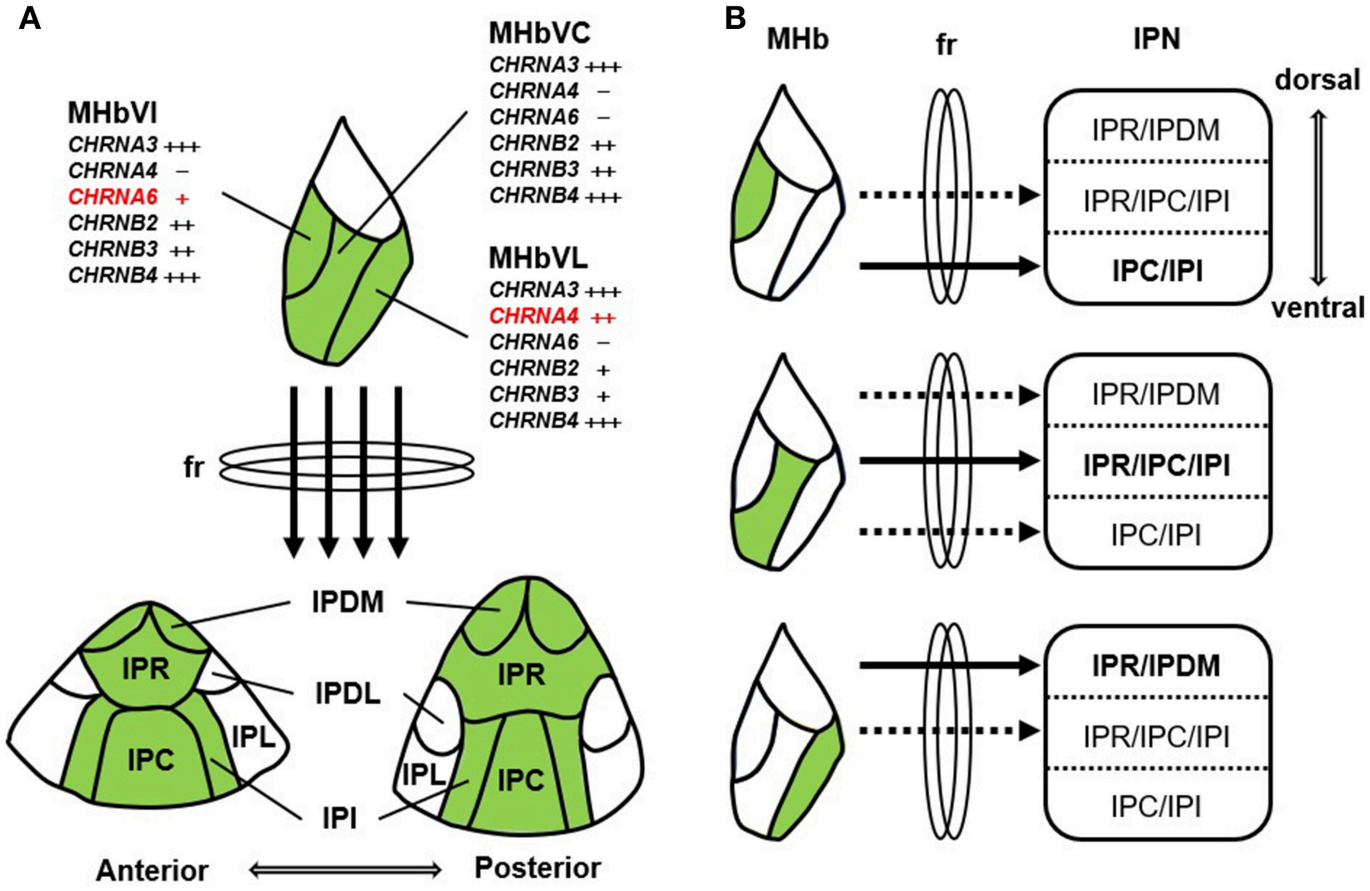
Schematic summary of nAChR subunit expression and the MHb-IPN pathway. **(A)** nAChR subunits are differentially expressed in the MHbV neurons, which project to central regions (IPDM, IPR, IPC, and IPI) excluding lateral regions (IPL and IPDL) of the IPN through the fasciculus retroflexus (fr). MHbV is composed of three subregions, such as inferior (MHbVI), central (MHbVC), and lateral (MHbVL). α6 nAChR subunit (*CHRNA6*) is exclusively found in the MHbVI. In contrast to *CHRNA6*, α4 nAChR subunit (*CHRNA4*) is mainly expressed in the MHbVL. IPN is composed of various subregions: rostral (IPR), dorsomedial (IPDM), dorsolateral (IPDL), caudal (IPC), and intermediate (IPI). **(B)** MHbVI, MHbVC, and MHbVL preferentially project to ventral, central, and dorsal regions of the IPN, respectively.

The MHbV, which contains the cholinergic neurons of the habenula, can be further subdivided longitudinally into inferior, central, and lateral subnuclei according to various anatomical, gene expression, and electrophysiological studies (5). This suggests that the cholinergic neurons present in these subnuclei may have distinct roles depending on their respective characteristics. Therefore, it will be necessary to identify the connections between neurons in the MHbV subnuclei and neurons in the various subnuclei of the IPN. In addition, Shih et al. revealed the precise localizations of nAChRs subunits in specific regions of MHb and IPN using immunohistochemistry and electrophysiology in the knock-in mouse strains expressing α3, α4, α6, β2, β3, and β4 subunit fused GFP, suggesting that despite their small size, neurons of the MHb have distinct neurophysiology, nAChR subunit expression, and sensitivity to nicotine (66). This study also indicated that nAChR-expressing cells of inferior, central, and lateral neurons in the MHbV project preferentially to ventral, central, and dorsal subnuclei of the IPN, respectively (Figure 1). Recently, Lima et al. also showed that the rostral part of the IPN connects with the central and lateral MHbV and the caudal part of the IPN connects with the central and inferior MHbV using retrograde tracing from the IPN to the MHb (67). Lima et al. also traced the efferent projections of the rat IPN using the anterograde tracing method and showed that the IPN makes a large number of connections with a variety of brain regions including the supramammillary nucleus, the septum/diagonal band complex, the temporal part of the hippocampus, the nucleus incertus, the laterodorsal tegmental nucleus (LDTg), and the caudal dorsal/median raphe nucleus (67). However, the results of this study were not confirmed by the retrograde labeling of IPN projecting sites. Quina et al. traced the afferent and efferent neural circuits of the IPN more finely using region-specific cre-expressing transgenic mice, anterograde viral tracer, and retrograde cholera toxin subunit b (CTb) tracer (68). After injecting the retrograde tracer CTb into several IPN projecting areas revealed by IPN anterograde tracing, such as hippocampus, septal nuclei, lateral hypothalamus, and lateral preoptic area, a comparison of CTb labeling with that of major immunohistochemical markers revealed that CTb labeling was not present in any IPN subnuclei receiving MHbD (SP) and MHbV (CHAT) inputs. Because there is no IPN-specific cre-transgenic mouse that can completely exclude the presence of neurons in surrounding structures, IPN descending sites cannot be easily observed with anterograde tracing without performing retrograde tracing. In addition, Ables et al. dissected the α5 nAChR subunit-expressing IPN neurons into two non-overlapping the α5^+^ cell populations containing highly enriched genes, Amigo1 and Epyc, both of which encode cell-surface adhesion proteins. α5-Epyc neurons project locally whereas α5-Amigo1 neurons send long projections to raphe and LDTg nuclei. Neuron-specific silencing via prevention of neurotransmitter release by cre-dependent membrane-tethered Ca^2+^-channel toxin showed that silencing Amigo1-cells prevented place preference for nicotine, whereas silencing Epyc-cells had no effect (69). This result suggests that although both α5-Amigo1 cells and α5-Epyc cells receive major inputs from the MHbV, only Amigo1 cells contribute the majority of projections from the IPN to the raphe and LDTg nuclei and functionally modulate the circuit responsible for behavioral changes to nicotine. In conclusion, more work will be required to elucidate each neural network that constitutes the cholinergic MHb–IPN–other brain regions axis and to study in detail the functions of each neural network and the axis as a whole.

As described above, habenula cholinergic neurons are closely tied to a variety of brain functions. Pharmacological approaches are needed to control the activity of these neurons. Interestingly, habenula cholinergic neurons express pharmacologically controllable GPCR proteins such as the GABA_B_ receptor and Tachykinin receptor 1 (TacR1). These ligand-dependent activity-modulating receptors may be useful targets for the treatment of various psychiatric disorders associated with malfunctioning habenula cholinergic neurons. For example, anhedonia-like behavior due to reduced habenular cholinergic signaling is not reversed by chronic administration of the standard antidepressant fluoxetine (21). Thus, enhancement of cholinergic signaling by direct activation of habenula cholinergic neurons may open the way for a new drug therapy for depression.

## Author Contributions

SY and JK led the literature search and HL drafted the manuscript. HK provided substantial contributions to the intellectual content of the manuscript. All authors approved the final version of manuscript to be published.

### Conflict of Interest Statement

The authors declare that the research was conducted in the absence of any commercial or financial relationships that could be construed as a potential conflict of interest.
